# Design of Electrochemical Sensor Based on Pumpkin Peel Biomass-Derived Carbon Black-Modified Electrode for the Detection of Lead Ions

**DOI:** 10.3390/s26051524

**Published:** 2026-02-28

**Authors:** Amal M. Aladwani, Esraa M. Bakhsh, Ekram Y. Danish, Zainab M. Hritani, Kalsoom Akhtar, Sher Bahadar Khan

**Affiliations:** Chemistry Department, Faculty of Science, King Abdulaziz University, P.O. Box 80203, Jeddah 21589, Saudi Arabiasbhkhan@kau.edu.sa (S.B.K.)

**Keywords:** electrochemical sensor, glassy carbon electrode, carbon black, cyclic voltammetry

## Abstract

This study reports a sustainable and experimentally simple electrochemical platform for monitoring trace Pb^2+^ using pumpkin peel-derived carbon black (CB) as a modifier on a Nafion-coated glassy carbon electrode (CB/Nafion-GCE). Agricultural waste pumpkin peels were converted into CB, offering a low-cost and environmentally friendly sensing material. CB produced at 650 °C was systematically characterized by SEM, TEM, EDX, XRD, FT-IR, and BET, revealing a mesoporous structure, high surface area, and partial graphitization that enlarged the electroactive area and reduced charge transfer resistance relative to the bare GCE. Under optimized square wave anodic stripping voltammetry (SWASV) conditions, the glassy carbon electrode modified with CB produced at 650 °C (CB650-GCE) exhibited a well-defined linear response towards Pb^2+^ with a limit of detection of approximately 0.19 µM and a limit of quantification of about 0.58 µM, together with good selectivity against common coexisting metal ions. The sensor also achieved satisfactory recoveries in tap and seawater samples, demonstrating its potential as a practical, green analytical tool for routine lead surveillance in environmental waters.

## 1. Introduction

Exposure to lead poses significant health risks and adversely affects multiple human organ systems. Lead can enter the human body through many sources, including lead-based products, contaminated soil, water, and air [1,2]. The primary mechanisms of lead toxicity involve the induction of oxidative stress and the disruption of critical biological functions [3,4,5]. The nervous system is particularly vulnerable, with severe neurological consequences such as encephalopathy, memory impairment, and attention deficits reported under both acute and chronic exposure conditions [5,6,7,8]. Due to its widespread environmental presence, often attributed to emissions from fuel combustion, lead contamination has become a global public health concern [9,10]. Consequently, the implementation of effective prevention and intervention strategies is essential for mitigating the detrimental health effects associated with lead exposure.

To address this issue, considerable effort has been devoted to developing reliable methods for detecting lead ions in water. Established analytical techniques include atomic absorption spectrometry, electrochemical methods, and spectrophotometry [11,12,13,14]. These approaches can achieve very low detection limits, down to approximately 0.1 μg L^−1^ for voltammetric and atomic absorption spectrometric techniques. Electrochemical detection of Pb^2+^ has attracted growing attention due to its inherent advantages of high sensitivity, relatively low cost, portability, and compatibility with on-site measurements [15,16,17,18,19,20,21].

Recent reviews of heavy-metal electrochemical sensors further highlight the progress made in improving selectivity, miniaturization, and applicability to complex water matrices [22,23,24]. Such developments are crucial for ensuring clean drinking water and safeguarding public health.

Beyond lead detection, carbon-based materials play a pivotal role in numerous applications owing to their unique physicochemical properties. Carbon, one of the most fundamental elements in nature, exhibits diverse characteristics resulting from its hybridized electronic orbitals (sp, sp^2^, sp^3^) and crystal anisotropy. This versatility enables the classification of carbon-based materials into zero-, one-, two-, and three-dimensional structures. Zero-dimensional forms include carbon quantum dots and fullerenes. One-dimensional structures consist of carbon fibers, carbon nanotubes, and nanowires. Graphene serves as a representative of 2D materials, while 3D structures encompass graphene foams and porous carbon frameworks [17,25]. This structural diversity underpins their broad use in energy storage, catalysis, environmental remediation, and chemical sensing.

Recently, carbon black (CB) has emerged as a particularly attractive nanomaterial for the development of electrochemical (bio)sensors. Its main advantages include high electrical conductivity, large specific surface area, chemical stability, and low cost [26,27]. Electrodes modified with carbon black typically exhibit rapid charge transfer kinetics, a high electroactive surface area, and performance comparable to that of graphene and carbon nanotubes. Moreover, nanostructured carbon black has been successfully employed for the simultaneous determination of lead and cadmium using differential pulse anodic stripping voltammetry [28,29,30], demonstrating its suitability for heavy-metal sensing.

In parallel, the development of sustainable, cost-effective, and highly sensitive electrochemical sensors for heavy-metal detection, particularly Pb^2+^, has become an active research focus. A prominent strategy involves the utilization of biomass-derived carbon materials, synthesized from diverse agricultural wastes, as advanced electrode modifiers. Plant-based residues such as sweetgum fruit, coconut shells, and other lignocellulosic materials have been converted into functional carbon materials and applied to trace Pb^2+^ analysis [31,32,33,34,35]. Despite this progress, ongoing research continues to emphasize the optimization of synthesis methodologies, structural characteristics, and sensor performance parameters. Critical factors influencing the electrochemical properties and analytical sensitivity of biomass-derived carbon sensors include the choice of biomass precursor, the specific carbonization conditions, and the electrode fabrication strategy. Recent comprehensive reviews underscore the importance of systematically tailoring these variables to maximize sensor efficacy [31,32,33,34,35]. In this context, there remains a need for studies that explicitly correlate carbonization temperature, microstructure, and surface chemistry of biomass-derived carbons with their analytical performance in Pb^2+^ detection, while simultaneously leveraging low-cost and locally available waste resources.

The electrochemical performance and analytical sensitivity of biomass-derived sensors are strongly influenced by factors such as the choice of biomass precursor, carbonization parameters, and electrode fabrication strategy. Recent studies emphasize the need for systematic optimization of these parameters to enhance sensor efficiency. In this study, pumpkin peels, an abundant yet underutilized agricultural product, were employed as a sustainable carbon precursor. By optimizing the calcination temperature, the obtained carbon material exhibited improved porosity, favorable surface functionalities, and enhanced electrical conductivity, contributing to efficient Pb^2+^ detection.

## 2. Experimental

### 2.1. Chemicals and Reagents

Cadmium chloride (CdCl_2_), chromium chloride (CrCl_3_), lead chloride (PbCl_2_), cobalt nitrate hexahydrate (Co(NO_3_)_2_·6H_2_O), and mercury chloride (HgCl_2_) (analytical grade, ≥99%) were obtained from BDH Chemicals Ltd. (Poole, UK) and Sigma-Aldrich (St. Louis, MO, USA). Glacial acetic acid (≥99.7% purity) and anhydrous sodium acetate (≥99%) were also acquired from these suppliers. Nafion^®^ 117 solution (5 wt.% in a mixture of lower aliphatic alcohols and water) was obtained from Sigma-Aldrich and used without further modification. Unless stated otherwise, all reagents were of analytical grade and employed as received. Ultrapure deionized water (18.2 MΩ·cm resistivity) was used in all solution preparations.

### 2.2. Synthesis of Carbon Black

**Scheme 1:**
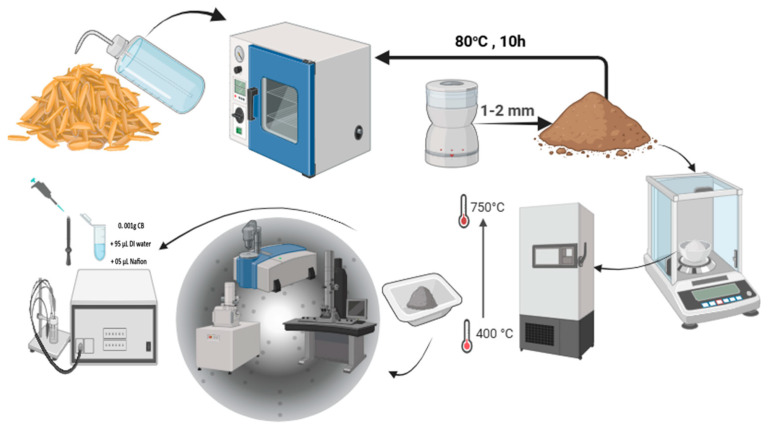
Preparation and sensing application of carbon black.

In Scheme 1, the preparation of pumpkin peel-derived carbon black (CB) begins with thorough washing of the fresh peels with distilled water to remove adhering soil, dust, and other surface contaminants, followed by oven-drying at 80 °C for 6 h to eliminate residual moisture and prevent hydrothermal degradation during subsequent processing. The dried peels were then mechanically ground to 1–2 mm pieces to increase surface area and ensure uniform heat and mass transfer, after which the powder is subjected to hydrothermal treatment at 80 °C for 10 h to leach out water-soluble phenolics and other low-molecular-weight organics that could interfere with carbonization or contaminate the final carbon material [30]. The pretreated biomass (10 g) is transferred to a tightly sealed crucible and placed in a tube furnace that is purged with high-purity N_2_ for 30 min and maintained under continuous N_2_ flow during heating to create an inert atmosphere that suppresses combustion and promotes controlled pyrolytic conversion of the organic matrix to carbon. Carbonization is carried out at selected temperatures (400–750 °C) to tune the textural and structural properties of the resulting CB; the optimized material obtained at 650 °C exhibits a favorable balance of mesoporosity, surface area, and partial graphitization. 

### 2.3. Apparatus

The surface morphology of carbon black was examined by capturing images at various magnifications using scanning electron microscopy (SEM) to observe the size and assess their morphological properties. Imaging was carried out using a Zeiss LEO Supra 55VP Field Emission SEM and a Zeiss 1530. To prepare the samples, the composite nanoparticle suspensions were diluted at a 1:10 ratio using a suitable dispersion medium. A droplet of the resulting dilution was then applied onto a polished aluminum stub for analysis, followed by vacuum drying. To improve conductivity and imaging clarity, the samples were coated with a thin gold layer using an EMITECH K450X sputter coater.

Transmission electron microscopy (TEM) analysis was conducted using a high-resolution TEM (JEOL Ltd., Akishima, Tokyo, Japan) operating at an accelerating voltage of 250 kV and a magnification of 20×. Prior to imaging, the CB samples underwent sonication using a probe under pulsed conditions (1 s intervals) at 85% amplitude, maintaining the temperature for 30 min. Subsequently, 50 µL of the sample was placed onto a TEM grid and allowed air dry for 5 h.

Elemental composition was analyzed by energy-dispersive X–ray spectroscopy (EDX) using an Oxford EDX system. Furthermore, the crystalline structure and phase identification of the integrated CB samples were characterized by X –ray diffraction (XRD) using a D8-Find diffractometer (Bruker, Madison, WI, USA) with CuKα radiation (λ = 1.5418 Å), operating at 40 mA and 40 kV with a step size of 0.01°. All samples were thoroughly dried before measurements and further processing utilizing a basic planetary ball mill (LZQM0.4L, Shicheng Desert Spring Mineral Gear Assembling Co., Ltd., Ganzhou, China), in which ball-treated steel balls of 0.1 cm diameter were used for processing for 1 h at 1500 rpm.

Surface area and pore size analyses were carried out using a Quantachrome NOVA touch 2LX surface area and pore size analyzer (Quantachrome Instruments, Boynton Beach, FL, USA). Samples were first degassed under vacuum at elevated temperature to remove contaminants and then cooled to cryogenic temperature using liquid nitrogen. Nitrogen gas served as an adsorbate and was introduced incrementally under vacuum. The volume of nitrogen adsorbed at each pressure was recorded to generate adsorption isotherms, from which the surface area and pore size distribution were calculated.

### 2.4. Modification of GCE

The glassy carbon electrode (GCE) modification was performed using a CB/Nafion composite. Initially, the surface of the GCE was mechanically polished with alumina powder to attain a mirror-like finish, followed by meticulous polishing and surface treatment steps to ensure optimal smoothness and reflectivity and thorough rinsing with deionized water and ethanol to remove any residual alumina particles. Subsequently, a CB suspension was prepared by dispersing 0.01 g of CB in 90 μL of deionized water with 10 μL of Nafion, followed by ultrasonication to achieve a uniform distribution of CB within the Nafion matrix. Subsequently, 5 μL of the well-dispersed solution was drop-cast onto the precleaned GCE surface. The electrode was then dried at room temperature under ambient conditions for approximately 30 min, ensuring the formation of a continuous, crack-free CB/Nafion film that uniformly covered the GCE surface and adhered strongly enough to withstand subsequent electrochemical measurements.

### 2.5. Electrochemical Measurements

A 0.2 M acetate buffer solution was introduced into a 10 mL electrochemical cell to serve as the supporting electrolyte. A standard three-electrode setup was utilized, comprising a glassy carbon electrode (GCE) as the working electrode (WE), a platinum wire as the counter electrode (CE), and an Ag/AgCl (3 M KCl) reference electrode (RE). The electrodes were carefully immersed in the electrochemical cell, ensuring proper electrical contact.

Electrochemical characterization was performed utilizing cyclic voltammetry (CV) and square wave anodic stripping voltammetry (SWASV) to detect and analyze the electrochemical behavior of lead ions. The obtained voltammograms were evaluated to determine the redox characteristics of Pb(II) and assess its interaction with the electrode surface, thereby enabling the establishment of a highly sensitive electrochemical detection approach.

The limit of detection (LOD) and limit of quantification (LOQ) were calculated according to IUPAC recommendations using the standard formulas LOD = 3.3σ/S and LOQ = 10σ/S, where σ is the standard deviation of the blank signal and S is the slope of the calibration curve obtained from SWASV measurements. All measurements were conducted on a single glassy carbon electrode modified with carbon black at 650 °C (CB650-GCE). For each concentration of Pb^2+^, triplicate measurements (n = 3) were performed under identical conditions to ensure data precision. The standard deviation of the blank was also obtained from triplicate measurements (n = 3). As such, the reported LOD and LOQ values reflect the repeatability of the sensor response using one electrode. Reproducibility studies using independently prepared electrodes are planned to further validate sensor performance.

Tap and seawater samples were collected in clean polyethylene bottles. To maintain optimal conditions for lead (Pb^2+^) analysis, the pH of each sample was adjusted to 3 using an acetate buffer. To assess the accuracy of Pb^2+^ detection, the standard addition method was used: several portions of each sample were spiked with known amounts of lead standard solution, while one portion was left unspiked as a control. All samples were analyzed for lead content using SWASV. The percentage recovery was calculated by comparing the measured concentrations to the amounts added. Each experiment was carried out in triplicate, and the results are presented as average values with their standard deviations.

## 3. Results and Discussion

### 3.1. Structural Characterization

#### 3.1.1. SEM, TEM and EDX

**Figure 1:**
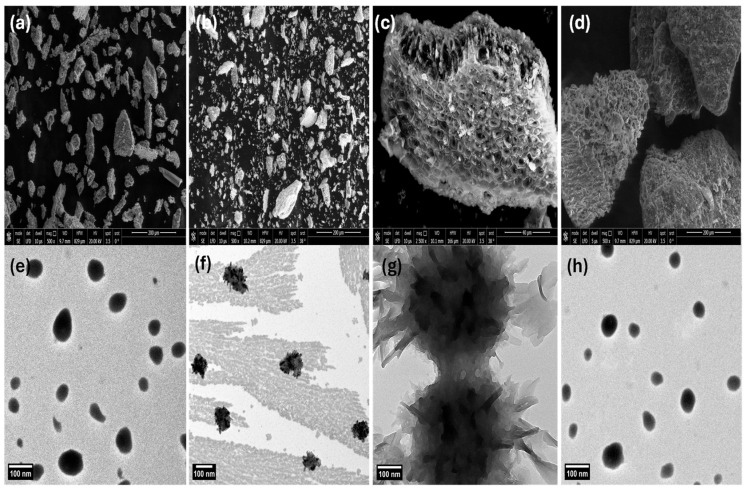
SEM images of CB prepared at (**a**) 750, (**b**) 700, (**c**) 650 and (**d**) 400 °C; TEM images of CB prepared at (**e**) 750, (**f**) 700, (**g**) 650 and (**h**) 400 °C, respectively.

The scanning electron microscopy (SEM) analysis reveals significant correlations between temperature variations and the structural properties of the CB samples. In Figure 1a, the sample subjected to the highest temperature exhibits smaller, fragmented particles with enhanced surface roughness and the presence of noticeable residues, indicating localized thermal or mechanical degradation. These morphological changes suggest a deviation from structural stability, potentially due to thermal decomposition and material deterioration. This aligns with observations by Havigh and Chenari [36], who reported significant particle breakdown and roughening of carbon fiber at elevated temperatures.

In contrast, the sample in Figure 1b, which also experienced a high-temperature environment, displays an uneven distribution of irregularly shaped particles with a rough surface texture. This morphology suggests a degree of thermal resilience, as the sample retains some structural integrity despite exposure to elevated temperatures.

A distinct honeycomb-like porous structure is observed in Figure 1c, corresponding to moderate-to-high temperature conditions. The increased porosity suggests thermal expansion, which is indicative of notable structural modifications induced by heat treatment. This transformation highlights the material’s dynamic response to thermal exposure.

Finally, the low-temperature condition is represented by the sample in Figure 1d, which exhibits a sponge-like structure characterized by larger voids and interconnected spaces. This morphology suggests greater structural stability at lower temperatures, with minimal thermal degradation and reduced material alterations. Zhang et al. [37] confirm that carbon black retains a porous and stable microstructure below 400 °C, showing only minor morphological changes.

Both our research and the findings of Kelesidis et al. [38] consistently demonstrate that temperature is the primary determinant in governing the morphological and porosity evolution of carbon black. At lower temperatures, carbon black maintains its structural integrity and original pore architecture. As thermal exposure increases, a marked progression of internal oxidation drives the formation and enlargement of pores, ultimately leading to highly porous or hollow morphologies. Under the most severe thermal conditions, oxidation is concentrated at particle surfaces, which promotes fragmentation and increased surface roughness while inducing only minor changes to internal porosity. This convergence of evidence affirms a robust, temperature-dependent pathway for carbon black transformation, confirming that observed microstructural changes are fundamentally coupled to porosity dynamics dictated by the thermal environment.

Figure 1e–h presents the transmission electron microscopy (TEM) images of the CB samples synthesized under varying thermal conditions, revealing notable differences in morphological and structural characteristics.

At 400 °C, carbon primarily appears as agglomerated particles, indicating a less organized structure with strong interparticle interactions. As the temperature increases to 650 °C, the carbon undergoes a transformation into spherical formations arranged in a sheet-like bundle, suggesting enhanced structural organization. At 700 °C, well-defined, distinct carbon sheets are observed, signifying a shift towards a more layered and separated morphology. At 750 °C, the black carbon remains uniformly dispersed, indicating improved stability and reduced particle aggregation.

Notably, while all samples exhibit some degree of structural degradation, the 650 °C-prepared black carbon retains a high degree of uniform dispersion, suggesting an optimal thermal condition for maintaining structural integrity. The stacking configuration of black carbon particles plays a pivotal role in determining their electrical properties, as well as their adsorption and desorption behaviors, which are critical for various electrochemical and catalytic applications.

**Figure 2:**
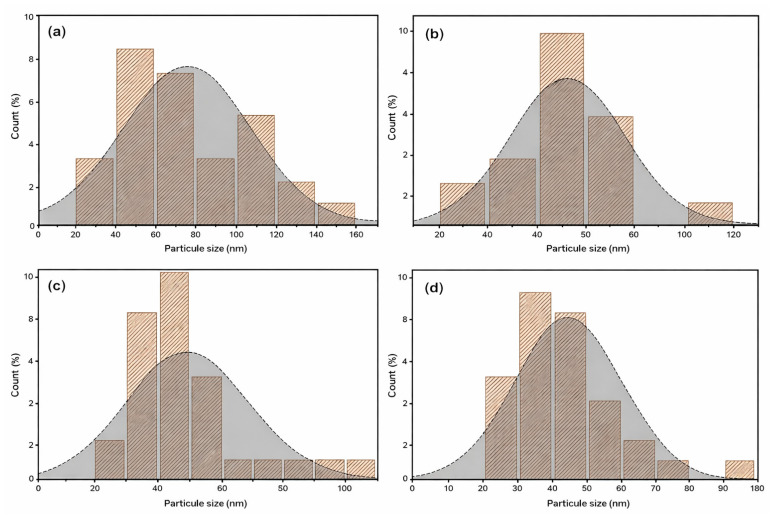
TEM particle size distribution analysis for (**a**) CB750, (**b**) CB700, (**c**) CB650 and (**d**) CB400.

TEM analysis revealed distinct particle size distributions (Figure 2) for samples calcined at different temperatures: sample (a) CB at 750 °C (CB750; 39.4 ± 2.41 nm), sample (b) CB at 700 °C (CB700; 26.7 ± 2.33 nm), sample (c) CB at 650 °C (CB650; 22.2 ± 2.17 nm), and sample (d) CB at 400 °C (CB400; 34.9 ± 2.81 nm). The relationship between calcination temperature and particle size exhibits a complex non-monotonic behavior, where intermediate temperatures (650–700 °C) produced the smallest particles, while both lower (400 °C) and higher (750 °C) temperatures resulted in larger particle sizes.

**Figure 3:**
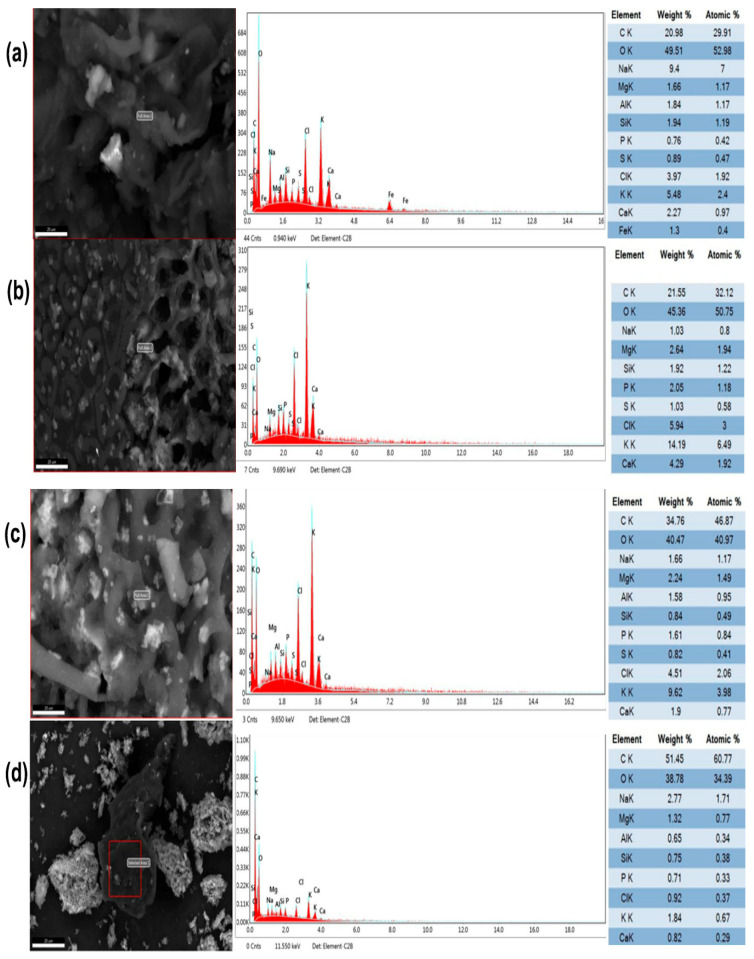
EDX spectra of (**a**) CB750, (**b**) CB700, (**c**) CB650 and (**d**) CB400.

Energy-dispersive X-ray spectroscopy (EDX) is a widely employed technique for elemental analysis, enabling the determination of sample composition (Figure 3). This technique is typically used in conjunction with scanning electron microscopy (SEM) to provide detailed microscopic and surface composition information.

In this study, EDX spectra were recorded for carbon black samples synthesized at 750, 700, 650, and 400 °C, revealing marked temperature-dependent changes in surface composition. The CB750 contained 20.98 wt% C and 49.51 wt% O, together with 9.40 wt% Na and 5.48 wt% K, and minor amounts of Mg, Al, P, Si, S, Cl, Ca, and Fe (<15 wt% in total), indicating a highly oxidized carbon matrix with significant inorganic residues. CB700 comprised 21.55 wt% C, 45.36 wt% O, 14.19 wt% K, 5.94 wt% Cl, and 4.29 wt% Ca, while CB650 exhibited 34.75 wt% C, 40.47 wt% O, 9.62 wt% K, and 4.51 wt% Cl, reflecting a moderate decrease in oxygen content and alkali/halide species. The lowest-temperature sample CB400 showed the highest C and O contents (51.45 and 38.78 wt%, respectively), with only 2.77 wt% Na as the major additional element, consistent with incomplete mineral removal at this stage. From these data, the C/O ratio decreases systematically with increasing calcination temperature, with values of 1.33, 0.86, 0.48, and 0.42 for CB400, CB650, CB700, and CB750, respectively, confirming that higher temperatures promote oxygen loss and a more mineral-rich surface. However, when these compositional trends are considered together with XRD and textural results, it becomes evident that the best electrical performance is not obtained from the sample with the highest C/O ratio (CB400), which suffers from limited structural ordering and persistent heteroatoms, but rather from CB650, where a moderate C/O ratio coincides with enhanced graphitic ordering and a more continuous carbon network. Therefore, the optimal electrochemical properties of the pumpkin-peel-derived carbon black arise from the combined effects of elemental composition, degree of graphitization, and pore structure, rather than from the C/O ratio alone, which is in agreement with previous studies [39,40,41] on carbonaceous electrode materials.

#### 3.1.2. XRD and FT-IR

The X-ray pattern of CB varies depending on temperature. Notable alterations in the 002 peak indicate that the structure of CB is considerably influenced by the extent of the calcination process [42,43].

**Figure 4:**
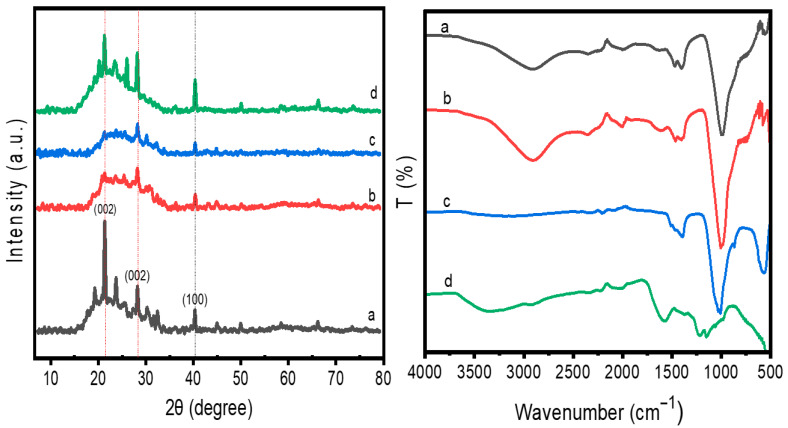
XRD and FT-IR spectra of (a) CB750, (b) CB700, (c) CB650 and (d) CB400.

The diffraction peaks observed in the pattern (Figure 4), particularly at 2θ ≈ 23° and 41°, correspond to the (002) and (100) planes of graphitic carbon, indicating a high degree of crystallinity. These results corroborate the findings of previous studies, such as [38,44]. As the calcination temperature decreases from 750 °C to 400 °C, the intensity and sharpness of peaks gradually diminish, exhibiting a broad, less defined pattern typical of amorphous carbon structures. The data support previous research indicating that lower calcination temperatures lead to less graphitization and increased structural disorder [45,46]. Among all, sample (c), calcined at 650 °C, presents a favorable balance between crystallinity and surface disorder, which enhances electrochemical activity.

The Fourier Transform Infrared (FT-IR) spectra of carbon materials synthesized at varying pyrolysis temperatures offer critical information regarding the structural and chemical characteristic transformations induced by thermal treatment. A key distinction is observed in the spectrum in Figure 4c (CB650) compared to the other spectra (a, b, and d), particularly in the fingerprint region (1500–500 cm^−1^), where the intensity and presence of functional group absorption bands reflect differences in graphitization and surface chemistry. The spectrum in Figure 4c exhibits prominent and well-defined absorption features in the 1000–500 cm^−1^ region, which are significantly more pronounced than Figure 4a,b, yet less diffused than Figure 4d. These peaks correspond to C–O stretching vibrations, associated with ether and epoxy groups, as well as out-of-plane C–H bending of aromatic rings, indicating that the spectrum in Figure 4c retains a partially oxygenated structure with a higher degree of disorder.

When compared to the spectrum in Figure 4a (750 °C) and the spectrum in Figure 4b (700 °C), the spectrum in Figure 4c displays a greater density of oxygen functionalities, suggesting a lower degree of graphitization and a more disordered structure. In contrast, the spectrum in Figure 4d (400 °C) is characterized by extensive oxygen functionalization and an amorphous nature, whereas the spectrum in Figure 4c represents an intermediate stage, demonstrating partial carbonization while preserving significant surface functionalities. Notably, the C=O stretching band near 1700 cm^−1^ is relatively strong in Figure 4c, weaker than Figure 4d but more pronounced than Figure 4a,b, indicating that carbonyl-containing groups remain partially preserved at this intermediate pyrolysis temperature.

Moreover, the strong C–O stretching bands observed in the 1000–1200 cm^−1^ region further confirms the presence of surface-bound oxygen moieties, which are substantially diminished in the higher-temperature spectra. The broadening and multiplicity of peaks below 1000 cm^−1^ suggest a higher degree of structural defects and disorder, reinforcing the classification of the spectrum in Figure 4c as an intermediate phase between the highly functionalized and amorphous characteristics of Figure 4d and the more graphitized structures of Figure 4a,b. This comparative analysis highlights the progressive removal of oxygen-containing functionalities and the increasing order in the carbon framework as the pyrolysis temperature rises, shedding light on the thermal evolution of carbonaceous materials.

Other studies have shown that carbon materials derived from different biomass sources can exhibit similar functional group signatures after pyrolysis [44,47,48], indicating comparable chemical environments on their surfaces. This similarity reinforces that the transformation during pyrolysis leads to convergence in surface chemistry regardless of the original feedstock.

#### 3.1.3. Surface Area and Pore Size Analyses

**Figure 5:**
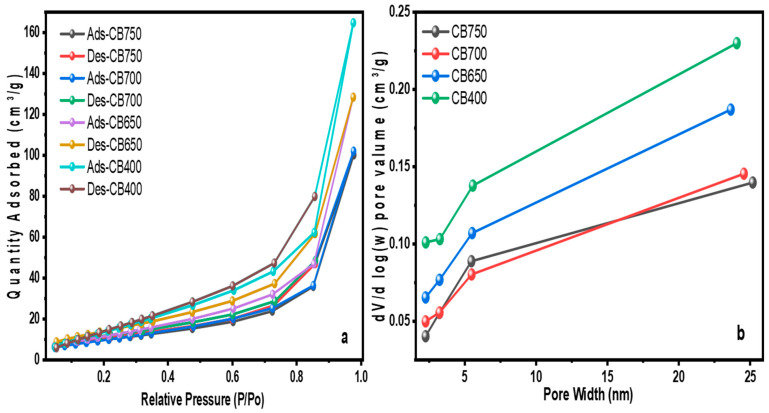
(**a**) BET nitrogen adsorption–desorption isotherms and (**b**) BJH pore size distribution of CB.

The comprehensive analysis of the nitrogen adsorption–desorption isotherms (Figure 5) and the quantitative pore structure data obtained from Brunauer–Emmett–Teller (BET) surface area and Barrett–Joyner–Halenda (BJH) pore size distribution measurements (Table 1) reveal a clear and systematic effect of carbonization temperature on the porous architecture and surface properties of the CB samples.

All samples exhibit type IV isotherms with pronounced hysteresis loops, confirming the dominance of mesoporous networks (Figure 5a). Notably, as the carbonization temperature decreases from 750 °C to 400 °C, both BET surface area and BJH pore volume undergo substantial enhancement. According to Table 1, the BET surface area increases from 37.2 m^2^/g at 750 °C to 65.8 m^2^/g at 400 °C, while the BJH cumulative adsorption pore volume rises from 0.1478 cm^3^/g to 0.2433 cm^3^/g over the same temperature range. This trend establishes that lower carbonization temperatures facilitate greater retention or development of accessible mesopores, increasing both the number and the volume of active adsorption sites.

Further insight is provided by the BJH pore size distribution curves (Figure 5b), where all samples exhibit a primary mesopore mode centered around 2–10 nm, indicative of a well-developed mesoporous structure ideal for molecular accessibility and rapid diffusion. The curves show that samples produced at lower temperatures (notably 400 °C) not only display higher overall pore volumes but also a greater proportion of larger mesopores (>10 nm), enhancing the hierarchical pore structure. This hierarchical architecture is highly advantageous in applications such as electrochemical sensing, where rapid analyte transport and high electrode–electrolyte contact area are critical for sensor performance.

Additionally, modest shifts in pore size distribution at different temperatures suggest a tunable balance between micro-, meso-, and microporosity. The desorption branch, particularly for the 650 °C and 400 °C samples, shifts toward smaller pore radius, which may indicate the presence of constricted or ink-bottle pores benefiting preconcentration mechanisms in sensors.

**Figure 6:**
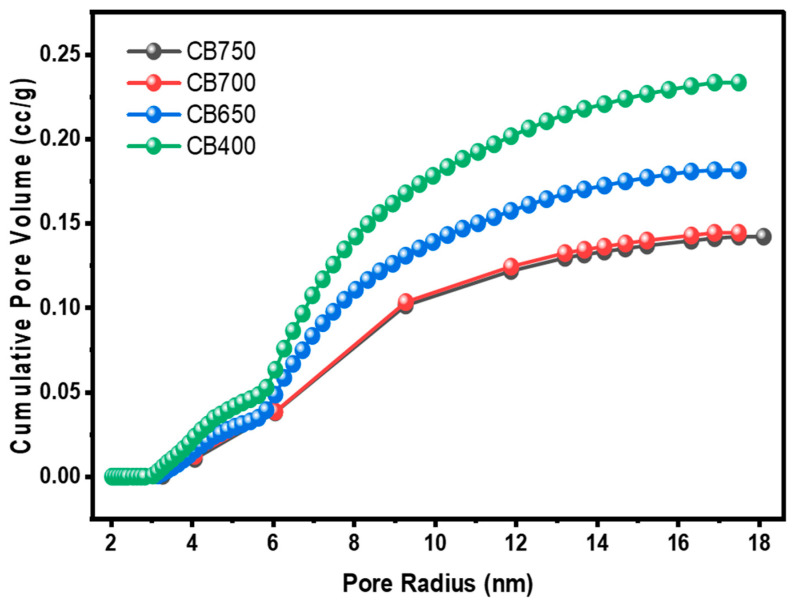
The cumulative pore volume versus pore radius for CB subjected to different temperatures.

Pore size distribution analysis, as supported by the cumulative pore volume data (see Figure 6), reveals that meticulous regulation of carbonization temperature has a pronounced impact on the development of mesoporous structures in CB materials. In particular, the CB650 exhibits a prominent pore mode at approximately 6.28 nm, which situates it firmly within the desirable mesoporous domain.

This precise pore architecture greatly facilitates essential processes such as rapid analyte diffusion and enhanced charge transfer efficiency, both of which are critical for high-performance electrochemical sensing applications.

A salient trend observed is that decreasing the carbonization temperature results in a progressive increase in cumulative pore volume [49,50]. CB650 demonstrates a significantly greater pore volume compared to CB samples subjected to higher temperatures (700 °C and 750 °C), while still maintaining robust structural integrity and circumventing the excessive microporosity often observed at far lower carbonization temperatures (e.g., 400 °C). This optimal balance enables CB650 to possess both a large accessible surface area and superior mechanical stability.

To reconcile the higher BET surface area of CB400 with the superior sensing performance of CB650, it should be emphasized that electrochemical activity is governed not only by accessible surface area but also by electrical conductivity and structural coherence: CB400, despite its larger area, is more amorphous and heteroatom-rich, whereas CB650 offers an optimal compromise between mesoporosity, graphitic ordering, and surface functionality, which translates into enhanced charge-transfer kinetics and improved Pb^2+^ detection.

The ability to fine-tune the carbonization process thereby affords direct control over the pore size distribution, pore volume, and interconnectedness of the CB material’s internal structure. The marked elevation in cumulative pore volume and the preferential formation of well-defined mesopores at 650 °C substantially increase the density of accessible active sites, facilitating swift analyte transport. These characteristics collectively contribute to improved analytical sensitivity and accelerate sensor response times. Moreover, the predominance of optimally sized interconnected mesopores ensures unimpeded analyte diffusion to electroactive sites, which is fundamental for achieving rapid signal generation and low detection limits.

In summary, the synergistic enhancement of specific surface area, mesopore volume, and structural coherence at optimized carbonization conditions underscores the pivotal role of process control in engineering carbon black for advanced sensor technologies. The CB650 sample exemplifies an ideal convergence of surface accessibility and mechanical durability, demonstrating how precise thermal treatment can yield carbon materials with superior functional properties for demanding electrochemical applications.

According to SEM, EDX, TEM, XRD, FT-IR, BET, BJH and pore size distributions, the synthesized product is best described as pumpkin peel-derived carbon black with partially graphitized domains and oxygenated surface groups, and it does not possess a unique molecular formula like a small organic or inorganic compound.

When moving from 400 to 750 °C, SEM/TEM images show that the carbon evolves from agglomerated, sponge-like particles with large voids into more compact, fragmented structures; the sample obtained at 650 °C exhibits uniformly dispersed nanosized particles with a honeycomb-like porous morphology that is particularly favorable for electrochemical applications. BET and BJH analyses reveal that decreasing the carbonization temperature from 750 to 400 °C increases both surface area (from 37.2 to 65.8 m^2^ g^−1^) and pore volume, while pore size distributions indicate that CB650 combines significant mesoporosity with good structural integrity, providing an optimal balance between accessible surface area and mechanical stability. XRD patterns show sharpening of the (002)/(100) peaks with increasing temperature, reflecting enhanced graphitization, whereas FT-IR spectra and EDX data demonstrate that higher temperatures progressively remove oxygen-containing surface groups and decrease the C/O ratio, so that CB650 retains a moderate density of oxygen functionalities together with improved graphitic ordering. These temperature-dependent trends mean that low temperatures favor high surface area but poor electrical ordering, high temperatures favor graphitization but reduce porosity and surface functionalities, and the intermediate 650 °C condition yields the most suitable combination of mesoporosity, electroactive surface area, and conductivity.

### 3.2. Electrochemical Detection Studies

#### 3.2.1. Electrochemical Behavior of Potassium Ferricyanide/Ferrocyanide: A Study Using CV and Electrochemical Impedance Spectroscopy (EIS) Techniques

To validate the electrochemical performance of the system, CV in a ferricyanide/ferrocyanide redox couple was carried out following the procedure described in reference. This standard test confirms that the electrochemical setup can perform voltammetric measurements with high precision [51].

All electrochemical measurements were performed using an analyzer in a three-electrode configuration, with GCE and the modified GCE functioning as the WE. The Ag/AgCl (3 M KCl) electrode acted as the RE, and a platinum wire was utilized as the CE. CV measurements were performed in a 0.1 M KCl solution containing 5 mM [Fe(CN)_6_]^3−^/^4−^ to evaluate the electrochemically active surface area of the electrodes, in accordance with the methodology described by Rashid et al. [52]. Electrochemical impedance spectroscopy was performed at a DC potential of 0.20 V vs. Ag/AgCl, using a sinusoidal AC perturbation of 10 mV over the frequency range from 100 kHz to 0.1 Hz.

**Figure 7:**
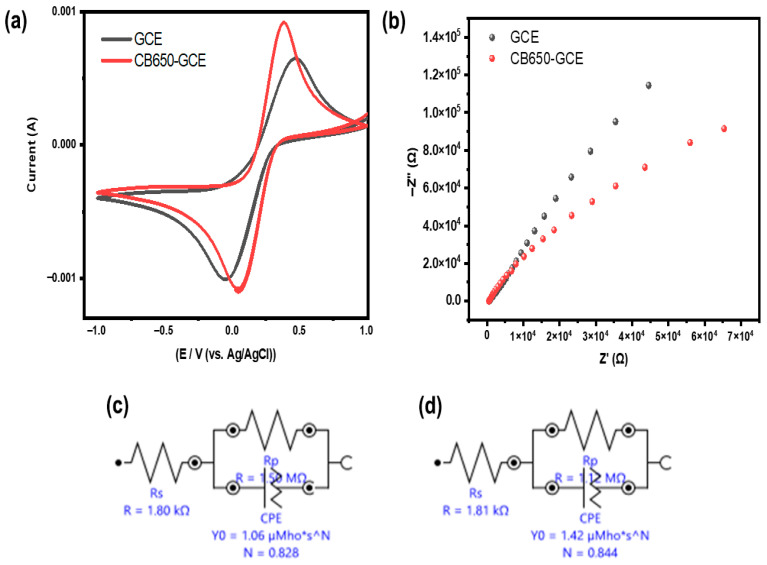
(**a**) Cyclic voltammograms obtained within the potential range of –1 V to +1 V, illustrating the redox behavior of 5 mM potassium ferricyanide in 1 M KCl as the supporting electrolyte on both uncoated (GCE) and coated (CB650-GCE) electrodes at a scan rate of 100 mV/s. (**b**) EIS spectra for both bare GCE and CB650-GCE in a buffer solution. (**c**,**d**) Equivalent circuit models representing (**c**) bare GCE and (**d**) CB650-GCE.

The electrochemical reduction of ferricyanide to ferrocyanide, followed by its oxidation, was performed on a bare GCE at a scan rate of 100 mV/s. A typical CV response for the [Fe(CN)_6_]^3−^/^4−^ redox couple is presented in Figure 7a, obtained from a 5 mM potassium ferricyanide solution in 1 M KCl. The comparison between the unmodified (black curve) and carbon black-modified (red curve) electrodes revealed a pronounced shift in both anodic and cathodic peak potentials. Additionally, the modified electrode exhibited a significant increase in peak current for both oxidation and reduction processes. These enhancements in electrochemical response indicate modifications in electron transfer kinetics and interfacial behavior, likely attributed to the increased surface area and conductivity provided by the carbon black layer on the GCE.

To quantitatively evaluate the electroactive surface areas, the Randles-Ševčík equation was applied to the ferricyanide/ferrocyanide redox couple:

*i*_p,c_= (2.69 × 10^5^) · *n*^3/2^ · *A* · *C*_0_ · *D_R_*^1/2^ · *ν*^1/2^

where $I_{p}$ is the anodic peak current, n=1, $D=7.6\times 10^{-6}$ cm^2^/s (diffusion coefficient of Fe (CN)_6_^3−^), c=5 mM, and ν=0.1 V/s (scan rate). The bare GCE exhibited an electroactive surface area of 0.066 cm^2^, which corresponds to approximately 93% of its geometric area (0.071 cm^2^), consistent with literature values for well-polished GCE [53,54]. The CB650-GCE showed a significantly larger electroactive area of 0.086 cm^2^, representing a 1.30-fold enhancement over the bare electrode. This increased electroactive surface area reflects the contribution of the porous carbon black film in providing greater electrode electrolyte contact area and more accessible active sites for electrochemical reactions.

Electrochemical Impedance Spectroscopy (EIS) was employed to further investigate the interfacial charge transfer properties of the electrodes. As illustrated in the Nyquist plot (Figure 7b), the impedance spectra of the bare GCE and CB650-GCE demonstrate distinct differences. The reduced semicircle diameter observed for the modified electrode indicates lower charge transfer resistance, confirming improved electron transfer efficiency at the electrode–electrolyte interface due to the carbon black coating (CB650), providing insight into its electrochemical performance. The real impedance component (*Z*′) represents resistive behavior, while the negative imaginary component (−*Z*″) reflects capacitive and inductive contributions. The bare GCE exhibits a larger semicircle, indicative of higher charge transfer resistance (*R*ct), due to its limited electroactive surface area and slower electron mobility. In contrast, the carbon black-coated electrode demonstrates a significantly reduced semicircle diameter, signifying enhanced electron transfer kinetics and lower *R*ct, attributed to the high conductivity and porous nature of carbon black. Additionally, the more pronounced linear tail at low frequencies suggests an increase in double-layer capacitance and improved mass transport properties at the electrode–electrolyte interface. These findings highlight that the CB650-GCE offers superior electrochemical performance, as evidenced by reduced charge transfer resistance, increased capacitance, and enhanced ion adsorption, indicating its effectiveness as a material for advanced electrochemical sensing platforms.

EIS analysis of the bare GCE and CB650-GCE provides critical insights into their interfacial charge transfer characteristics. The equivalent circuit models presented in Figure 7c,d illustrate key electrochemical parameters, including solution resistance (*R*s), charge transfer resistance (*R*ct), and constant phase element (CPE) values. The solution resistance for the bare electrode was measured at 1.80 kΩ, whereas the coated electrode exhibited a slightly higher Rs of 1.81 kΩ, indicating minimal impact of the coating on the electrolyte resistance. However, a significant reduction in charge transfer resistance was observed upon modification, with *R*p decreasing from 1.50 MΩ for the bare GCE to 1.12 MΩ for the coated electrode. This reduction suggests that the coating facilitates improved electron transfer kinetics, thereby enhancing the overall electrochemical performance. Furthermore, the CPE parameter Y0 increased from 1.06 in the bare electrode to 1.42 in the coated electrode, implying an increase in surface capacitance, likely due to enhanced electroactive surface area or improved charge storage capability. Additionally, the increase in exponent *N* from 0.828 to 0.844 indicates a shift toward more ideal capacitive behavior, reinforcing enhanced conductivity and charge distribution at the electrode interface. These findings collectively demonstrate that the electrode modification substantially improves charge transfer efficiency, making the coated electrode a more effective platform for electrochemical applications, such as sensing and electrocatalysis.

#### 3.2.2. Electrochemical Investigations on Pb^2+^ Using CV

**Figure 8:**
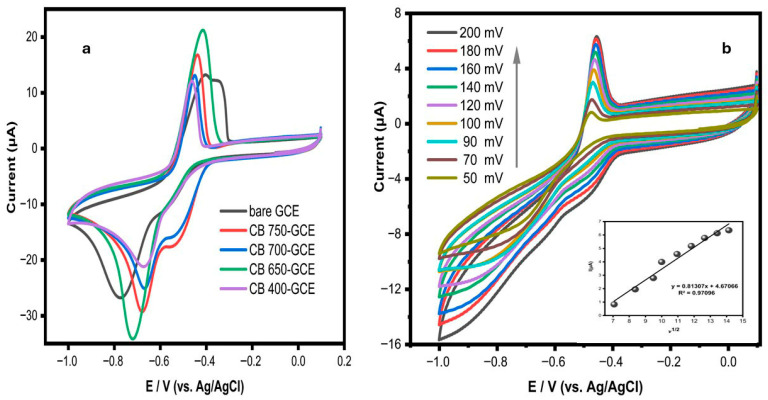
(**a**) Cyclic voltammogram of 100 µL Pb^2+^ ($4.8\times 10^{-3}$ M) in 0.2 M acetate buffer (pH 3.0) at CB-GCE vs. Ag/AgCl. (**b**) CB650-GCE CVs of 100 µL PbCl_2_ ($4.8\times 10^{-4}$ M) at different scan rates (−0.1 to 0.1 V), showing a linear relationship between anodic peak current (*I*pa) and the square root of scan rate ${\left(\;\upsilon \right)}^{1/2}$.

In Figure 8a, the current responses of the carbon black-coated electrodes are significantly higher than those of the GCE, indicating enhanced charge transfer efficiency and improved electrochemical kinetics due to the carbon black modification. Notably, the electrode modified with CB650 displays the most pronounced oxidation and reduction peaks, indicating a highly reversible redox reaction. This observation implies that the 650 °C prepared carbon film electrode (CB/Nafion) demonstrates the highest sensitivity for the electrochemical detection of lead (Pb^2+^) ions.

To further explore the effect of pH on the electrochemical performance of lead ion detection, CV measurements were performed in solutions containing 100 µL of Pb^2+^ at pH levels of 3, 4, and 6 using CB650-GCE. The results indicate that at pH 6, the redox peak currents are significantly lower, suggesting reduced electron transfer efficiency at this pH. Conversely, the highest redox peak current is achieved at pH 3.0, indicating that this condition is optimal for detecting lead ions.

The acetate buffer (pH 3.0) serves as the electrolyte, creating a stable ionic environment that is crucial for redox reactions. The buffering capacity of acetate ions mitigates significant pH variations at the boundary between the electrode and the electrolyte, which could otherwise hinder the redox activity of Pb^2+^ or disrupt the catalytic characteristics of the CB/Nafion film [52]. Additionally, the ionic strength and buffering capabilities of the solution enhance the stability of the electrical double layer, facilitating efficient electron transfer and optimizing the electrochemical response [55,56].

Limited complexation of Pb^2+^ by acetate was a key reason for fixing the buffer concentration at 0.2 M. At higher acetate concentrations, Pb^2+^ increasingly forms Pb–acetate complexes (e.g., Pb(CH_3_COO)^+^, Pb(CH_3_COO)_2_), which lowers the activity of free Pb^2+^ in solution and thus decreases the electrochemically active fraction that can be reduced at the electrode surface, leading to weaker analytical signals. In contrast, using much lower acetate concentrations would reduce the solution’s ionic strength and buffering capacity, increasing solution resistance (iR drop) and making the pH at the electrode less stable during measurements, which also degrades peak shape [57].

In this optimized configuration, the integration of CB650/Nafion and acetate buffer establishes a highly sensitive and stable electrochemical environment, significantly enhancing the performance of the modified GCE for accurate and reproducible lead ion detection.

Valuable insights into electrochemical mechanisms can often be obtained by analyzing the relationship between peak current and scan rate. To this end, the voltammetric behavior of CB was examined across a range of scan rates using CV. This scan rate analysis was conducted to determine whether the electrochemical process occurring at the CB650-GCE is controlled by diffusion or adsorption. A linear relationship was observed between the peak anodic current (*I*pa) and the square root of the scan rate (*υ*^1/2^) over the range of 50–200 mV/s, suggesting that the process is diffusion-controlled. This behavior can be mathematically expressed as follows:

$$I\mathrm{pa}\;=\;0.81307\;\upsilon^{1/2}\;+\;4.67066,\;R^{2}=0.97096$$

The influence of scan rate in CV on the detection of Pb^2+^ ions is paramount, as it directly affects both the peak current and the morphology of the voltammogram. An increase in scan rate typically results in an enhancement of the peak current due to kinetic factors associated with mass transport and electron transfer dynamics. Specifically, in the context of Pb^2+^ detection, elevated scan rates can yield sharper peaks, which signify enhanced electrochemical activity. However, excessively high scan rates may induce peak overlap and diminished resolution, thereby complicating data interpretation [42].

In examining the effect of scan rates ranging from 50 mV/s to 200 mV/s, it becomes evident that a scan rate of 100 mV/s is optimal for achieving reliable results. This scan rate effectively balances the need for sufficient mass transport with the prevention of excessive peak broadening.

Furthermore, the sweep rate influences the reversibility of the electrochemical reaction. At lower scan rates, the system may attain a quasi-equilibrium state, facilitating more precise quantification of Pb^2+^ concentration. In contrast, higher scan rates may hinder equilibrium attainment, resulting in a kinetically controlled process that could misrepresent the actual concentration of lead ions.

#### 3.2.3. Electrochemical Detection of Pb^2+^ Using SWASV

SWASV is an electrochemical approach that is preferred in many applications due to its quick analysis, high sensitivity, and low oxygen interference [58]. By combining square-wave modulation and a staircase potential, SWASV makes it easier to study both quick and slow electrode reactions [59]. More advanced versions, such as Fourier transform SWV, provide more thorough data than traditional alternating current voltammetry (ACV) by enabling the evaluation of electrode admittance at various harmonic frequencies [60]. Its usage in environmental monitoring and heavy metal investigations has expanded due to recent advancements that have improved its analytical capabilities [61].

The sensitivity of the technique is mostly determined by the peak current, an essential electroanalysis parameter. Peak current enhancement is closely related to electrode kinetics, which is impacted by several operating parameters, including modulation amplitude, frequency, deposition potential, and deposition time [62]. These variables affect how the electrode behaves dynamically during square wave voltammetric scans.

SWASV creates discrete potential steps by superimposing square wave pulses onto a staircase potential. By reducing capacitive currents and background noise, this technology improves the identification of electroactive species, boosting the electroanalytical technique’s sensitivity and precision.

In this study, we systematically examined several parameters affecting the performance of SWASV for the detection of lead ions (Pb^2+^) using a CB650-GCE.

**Figure 9:**
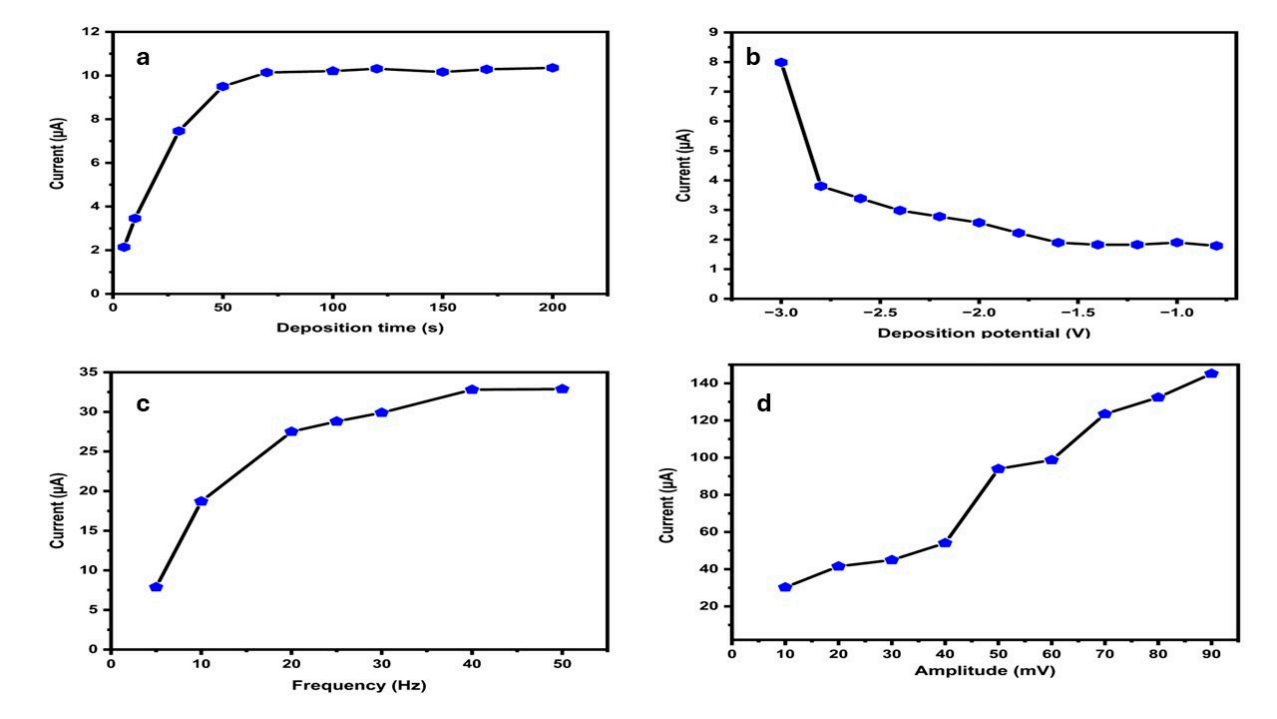
(**a**) The influence of deposition duration on peak current, (**b**) the impact of deposition potential, (**c**) the role of operating frequency, and (**d**) the effect of pulse amplitude on peak current using SWASV. The graphs were obtained using SWASV (3.6 μM Pb^2+^) in acetate buffer (pH 3.0).

Initially, we optimized the exposure time of the CB650-GCE in an acetate buffer (pH 3.0) containing 3.6 μM Pb^2+^, applying a holding potential of −0.8 V versus Ag/AgCl. The effect of deposition time, varied from 0 to 200 s, was evaluated via potential sweeps at a frequency of 25 Hz and a pulse amplitude of 0.02 V. As illustrated in Figure 9a, the peak current increased markedly with deposition time, reaching a plateau at around 70 s, beyond which a stable and analytically significant signal was obtained. This outcome represents a notable enhancement in efficiency compared to conventional cyclic voltammetry, thus demonstrating the method’s suitability for routine analysis.

Next, we investigated the influence of deposition potential, ranging from −0.8 V to −3.0 V, on the SWASV peak current. Although –3.0 V yielded the highest peak current (Figure 9b), −0.8 V was selected as the optimal deposition potential for all subsequent analytical measurements because it provides excellent sensitivity with improved signal stability and avoids potential secondary electrochemical reactions at more negative potential, ensuring better reproducibility and long-term electrode stability.

Further optimization involved studying the effect of operating frequency on the SWASV response, as depicted in Figure 9c. Peak currents increased with frequency, reaching a maximum at 50 Hz, which was therefore identified as the optimal frequency. Lastly, we examined the impact of pulse amplitude (10–90 mV) on peak current in the presence of 3.6 μM Pb^2+^. As illustrated in Figure 9d, the current response improved with increasing amplitude and produced larger peak currents. They also led to noticeable peak broadening and a slight shift in the peak potential, indicating increased non-Faradaic contributions and partial loss of resolution. In contrast, an amplitude of 50 mV provided a well-defined, symmetric stripping peak with a favorable signal-to-noise ratio and minimal peak distortion and was therefore selected as the optimal compromise between sensitivity and peak morphology.

To optimize the SWASV approach for the sensitive detection of lead ions, our results together highlight the crucial interactions among exposure time, deposition potential, operating frequency, and pulse amplitude, creating a strong foundation for future electroanalytical applications.

Measurements were conducted over a potential range of −0.7 V to 0.0 V, with a deposition time of 70 s, deposition potential of −0.8 V, modulation amplitude of 0.05 V, and frequency of 50 Hz. The corresponding calibration curve demonstrates a linear relationship between peak current and Pb^2+^ concentration.

The SWASV response of CB650-GCE was systematically evaluated across varying concentrations of lead ions (Pb^2+^). The concentration range investigated spanned from 0.29 μM to 1.09 μM, within an applied potential window of −0.7 V to 0.0 V. The resulting voltammograms exhibited distinct, well-defined peaks corresponding to the electrochemical reduction of Pb^2+^, demonstrating the high sensitivity of the CB-modified electrode.

Key experimental parameters were optimized to enhance signal quality. These conditions were selected to maximize preconcentration efficiency and improve charge transfer kinetics, thereby enhancing the analytical performance of the sensor.

**Figure 10:**
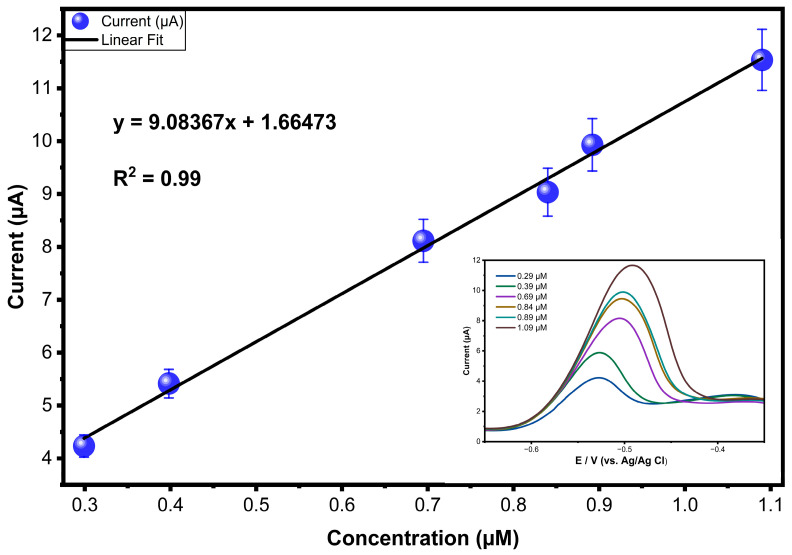
SWASV of CB650-GCE recorded at varying Pb^2+^ concentrations (0.29–1.09 μM).

As shown in the inset of Figure 10, the calibration curve exhibits a strong linear relationship between peak current (I, μA) and Pb^2+^ concentration (μM).

*I* (μA) = 9.08367 (Pb^2+^) *+* 1.66473.

with a correlation coefficient (R^2^) of 0.99, confirming excellent linearity, reproducibility, and sensor reliability.

The calculated limit of detection (LOD) and limit of quantification (LOQ) were 0.19 µM and 0.58 μM, respectively, indicating the method’s capability to detect trace levels of lead ions with high sensitivity. Furthermore, the sensitivity of the sensor, derived from the slope of the calibration curve, was found to be 9.08367 μA μM^−1^. This high sensitivity highlights the efficacy of the CB650-GCE as a promising platform for the selective and sensitive detection of Pb^2+^ in environmental and analytical applications. Table 2 provides a concise summary of recent publications on electrochemical methods used for detecting lead ions. For each Pb^2+^ concentration, three replicate SWASV measurements were recorded, and the data points in the calibration curve correspond to the mean peak current, with error bars representing the standard deviation (n = 3).

Table 2 summarizes the analytical performance of various modified electrodes reported for Pb^2+^ determination and positions the CB650-GCE sensor developed in this study within the current state of the art. The listed methods span different voltammetric techniques, including DPASV, DPV, SWV, and SWASV, and show limits of detection ranging from the sub-nanomolar level up to tens of micromolar, depending on the modification strategy and measurement protocol. While some systems based on highly specialized architectures, such as poly(riboflavin)/CB/GCE, achieve extremely low LOD values, they often involve more complex fabrication routes and less accessible materials. In contrast, our CB650-GCE (this study), operating via SWASV, attains an LOD of 0.19 µM, which is comparable to or better than several reported SWASV sensors (e.g., AuNPs-L1/SPCE, AuNPs/CNFs, and PEDOT/NTA), while relying on a simpler, carbon-based modification. This comparison indicates that the proposed CB650-GCE platform offers a competitive balance between sensitivity and practical simplicity, making it an attractive alternative for routine lead ion monitoring.

**Scheme 2:**
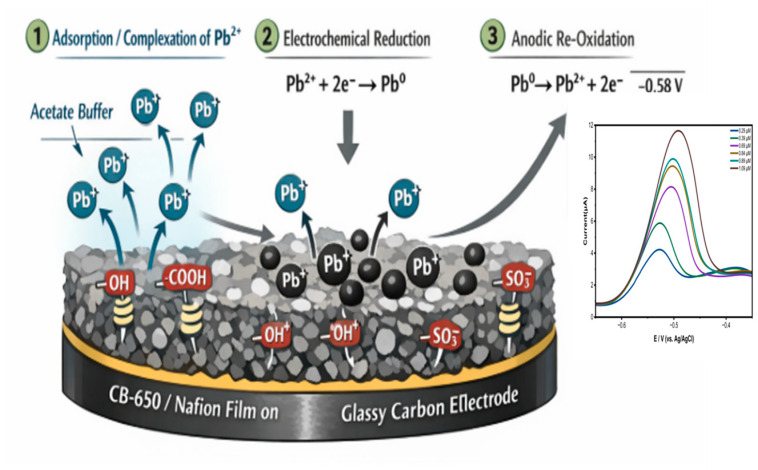
Schematic illustration of Pb^2+^ sensing mechanism at the CB650/Nafion-modified GCE.

To clarify the sensing mechanism, Scheme 2 summarizes the proposed sequence of events occurring at the CB650/Nafion/GCE surface during SWASV detection of Pb^2+^. In the first step, Pb^2+^ ions from the acetate buffer diffuse into the mesoporous CB650/Nafion film and are preconcentrated through electrostatic attraction and complexation with surface oxygenated groups (–OH, –COOH) on the carbon black and the sulfonate (–SO_3_^−^) groups of Nafion. Upon application of the cathodic SWASV potential, the adsorbed Pb^2+^ species are electrochemically reduced to metallic Pb^0^ on the conductive carbon surface (Pb^2+^ + 2e^−^ → Pb^0^), benefiting from the good electronic connectivity and high electroactive area of the CB650 film. During the forward pulse, Pb^0^ is re-oxidized to Pb^2+^, generating the anodic SWASV peak at approximately −0.58 V, which is used for quantitative determination of lead. The synergistic combination of efficient Pb^2+^ preconcentration by the functionalized CB/Nafion matrix and rapid electron transfer through the partially graphitized carbon network accounts for the high sensitivity of the sensor.

#### 3.2.4. Applicability of CB650-GCE in Pb^2+^ Detection

##### Selectivity

The CB650-GCE in the electrochemical detection of Pb^2+^ was rigorously evaluated by investigating its response under the influence of coexisting metal ions, which may interfere; Hg^2+^, Cd^2+^, Co^2+^, and Cr^3+^ were selected as potential interferents because they are among the most relevant toxic or regulatory heavy metals commonly encountered together with Pb^2+^ in contaminated natural and industrial waters, and they are frequently monitored in environmental control and drinking-water quality studies. Furthermore, Cd^2+^ and Hg^2+^ are classical co-analytes in voltammetric heavy-metal determinations, while Co^2+^ and Cr^3+^ are typical transition-metal pollutants from industrial effluents; their inclusion therefore allows direct comparison of the selectivity of the present sensor with previously reported Pb^2+^ sensors in the literature.

Selectivity was quantitatively evaluated from the individual SWASV responses of Pb^2+^, Cd^2+^, Hg^2+^, Co^2+^, and Cr^3+^ recorded with the CB650-GCE in the Pb^2+^ peak potential window (− 0.65 to − 0.45 V). At the characteristic Pb^2+^ peak potential, the stripping current of Pb^2+^ was used as the reference signal, and the interference from each coexisting ion was expressed as:

$$\%\mathrm{interference}=I_{\mathrm{interferent}\;\mathrm{at}\;E_{\mathrm{Pb}}}/I_{\mathrm{Pb},\mathrm{peak}}\times 100$$

The calculated interference values for Cd^2+^, Hg^2+^, Co^2+^, and Cr^3+^ were all low (typically below ≈10%), indicating that these ions contribute only minor background currents at the Pb^2+^ peak potential. Thus, within the investigated conditions, only Pb^2+^ generates a pronounced stripping peak in this potential window, confirming the high selectivity of the CB650-GCE toward Pb^2+^ and its suitability for reliable determination of lead in the presence of other common metal ions.

**Figure 11:**
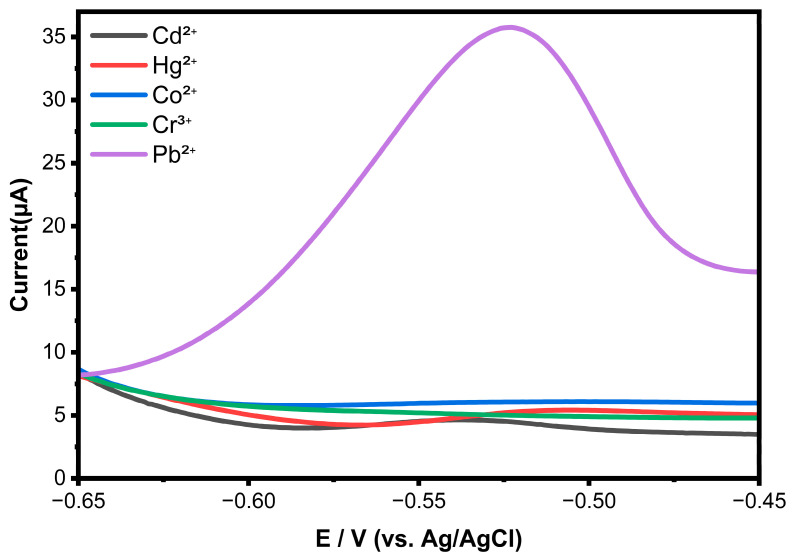
Individual SWASV responses of the CB650-GCE toward 100 µM Cd^2+^, Hg^2+^, Co^2+^, Cr^3+^, and Pb^2+^ in 0.2 M acetate buffer (pH 3.0) within the Pb^2+^ peak potential window (−0.65 to −0.45 V), showing that only Pb^2+^ produces a pronounced stripping peak while the other metal ions give only minor background currents.

Figure 11 presents the individual SWASV responses of each metal ion within a narrower potential window −0.65 to −0.45 V). In this region, only Pb^2+^ generates a significant stripping peak, whereas Cd^2+^, Hg^2+^, Co^2+^, and Cr^3+^ produce only minor background currents, confirming the high selectivity of the CB650-GCE toward Pb^2+^ and its suitability for selective lead determination in complex matrices. When compared with previously reported Pb^2+^ sensors based on ion-imprinted polymers, bismuth nanostructures, or gold-nanoparticle-modified electrodes, the CB650-GCE offers comparable, and in some cases superior, selectivity while relying on a simpler and more economical fabrication route, which is advantageous for practical applications. Overall, the remarkable selectivity observed here highlights the potential of the CB650-GCE for trace-level Pb^2+^ detection in industrial and environmental samples, where minimizing interference from other metal ions is essential; nevertheless, more extensive selectivity studies involving additional relevant interferents such as Cu^2+^ and Ag^+^, as well as mixed-ion conditions, are still required to fully establish the sensor’s performance under realistic field scenarios.

##### Application for Real Samples

To evaluate the analytical performance of the proposed method for the determination of Pb^2+^ in real water samples, both sea water and tap water were analyzed using the standard addition approach (Figures S2 and S3). As shown in Table 3, recovery experiments were performed by spiking samples with known concentrations of Pb^2+^ (4.35 and 5.80 μM). For sea water, recoveries of 124.79 ± 1.76% and 100.00 ± 3.83% were obtained for the respective spiking levels, indicating the method’s accuracy and the possible presence of matrix effects at lower spiking concentrations. Similarly, tap water samples exhibited recoveries of 107.22 ± 1.25% and 100.00 ± 0.33% for 4.35 and 5.80 μM additions, respectively, demonstrating excellent agreement between added and found values. Notably, the small Pb^2+^ peak observed in the unspiked (pure) tap water voltammogram arises from the native lead content of the sample, which is quantified by extrapolation of the standard-addition plot rather than from any added standard. The consistently low relative standard deviations for all measurements confirm the good precision of the procedure, supporting the applicability of the proposed method for reliable Pb^2+^ determination in different water matrices. The relative standard deviations (RSD) for all measurements were low, confirming the precision and reproducibility of the proposed method. These results suggest the method is suitable for the accurate determination of Pb^2+^ in various water matrices.

The slightly elevated recovery in seawater at the lower spiking level (124.79 ± 1.76%) may reflect ionic strength or complexation effects specific to the marine matrix; however, recovery at the higher level (100.00 ± 3.83%) and the consistent results in tap water (100.00 ± 0.33%) confirm that the method is robust and reliable for practical environmental monitoring. This study did not include validation against a standardized reference method such as ICP-MS; future work will involve comparison with ICP-MS and certified reference materials to confirm method trueness.

## 4. Conclusions

In this study, pumpkin peel waste was successfully transformed into a family of carbon black materials by nitrogen-assisted carbonization at temperatures between 400 and 750 °C, and the resulting carbons were systematically evaluated as electrode modifiers for Pb^2+^ detection. Comprehensive physicochemical characterization by SEM/TEM, EDX, XRD, FT-IR, and BET/BJH demonstrated that the carbon structure evolves from highly oxygenated, amorphous, high-surface-area solids at low temperatures to more graphitized, less porous materials at high temperatures. Among the prepared samples, the material obtained at 650 °C (CB650) provides a favorable compromise, combining a well-developed mesoporous network, enhanced graphitic ordering, and a moderate density of oxygen-containing surface groups.

When integrated into a Nafion-assisted CB650-modified glassy carbon electrode (CB650-GCE) and operated under optimized square-wave anodic stripping voltammetry conditions, this material enabled sensitive and reliable detection of Pb^2+^ in acidic acetate buffer. The CB650-GCE exhibited a linear response in the low-micromolar range, with a correlation coefficient close to unity and limits of detection and quantification of approximately 0.19 µM and 0.58 µM, respectively.

These performance metrics are comparable to, or better than, those reported for several more complex Pb^2+^ sensing architectures, while relying on a simple one-step drop-casting procedure and an inexpensive, renewable biomass precursor. The sensor also delivered satisfactory recoveries for Pb^2+^ in spiked tap and seawater samples, highlighting its potential for practical environmental monitoring.

Beyond the specific application to lead detection, the results establish a clear structure–property performance relationship linking biomass precursor treatment temperature, carbon microstructure and surface chemistry, and electroanalytical response. This provides a rational framework for designing biomass-derived carbon materials tailored for electrochemical sensing.

## Figures and Tables

**Table 1:** BET surface area and BJH pore volumes of CB samples prepared at different temperatures.

Temperature (°C)	BET Surface Area (m^2^/g)	BJH-Adsorption (cm^3^/g)	BJH-Desorption (cm^3^/g)
**750**	37.2	0.1478	0.1492
**700**	39.9	0.1497	0.1546
**650**	47.5	0.1901	0.1961
**400**	65.8	0.2433	0.2589

**Table 2:** Comparison of electrochemical sensors designed for lead ions using various modified electrodes.

Modification/Platform	Method	LOD (μM)	LOQ (μM)	Linear Range (μM)	Sensitivity (μA μM^−1^)	Notes on Selectivity/Matrix
poly(riboflavin)/CB/GCE [63]	DPASV	0.00013	0.0004	0.001–1.0	18.5	Common metal ions
BF-PPy/UIO-66-NH_2_/GCE [64]	DPV	0.003	0.01	0.01–5.0	12.3	Water samples
AuNPs-L1/SPCE [65]	SWASV	0.298	0.9	0.5–50	0.42	Simultaneous Pb^2+^/Cd^2+^
PEDOT/NTA [19]	SWV	11.2	34	5–100	-	Interference from Cu^2+^
GrRAC-70% [66]	DPASV	0.8	2.4	0.5–25	3.2	River water
AuNPs/CNFs [67]	SWASV	0.25	0.76	0.1–10	5.8	Drinking water
AuNP-CNT [68]	SWASV	0.00263	0.008	0.005–1.0	22.1	Selective vs. Cd^2+^, Zn^2+^
MWCNT tower based GCE [69]	SWASV	0.012	0.036	0.05–10	8.9	Environmental water
thiol functionalized clay/CPE [70]	SWASV	0.06	0.18	0.2–15	4.5	Tap water
CB650-GCE (This study)	SWASV	0.19	0.58	0.29–1.09	9.08	Cd^2+^, Hg^2+^, Co^2+^, Cr^3+^ tested

**Table 3:** Analytical results of Pb^2+^ detection in different water samples by the proposed method.

Sample	Added (μM)	Found ^a^ (μM) ± SD	Recovery ^a^ ± RSD (%)
	0.0	-	-
Sea water	4.35	5.42 ± 0.077	124.79 ± 1.76
	5.80	5.80 ± 0.222	100.00 ± 3.83
	0.0	-	-
Tap water	4.35	4.67 ± 0.058	107.22 ± 1.25
	5.80	5.80 ± 0.019	100.00 ± 0.33

^a^ The average of measurements (n = 3).

## Data Availability

Data will be available upon request.

## Supplementary Materials

The following supporting information can be downloaded at: https://www.mdpi.com/article/10.3390/s26051524/s1, Figure S1: (a) The influence of deposition duration on peak current, (b) the impact of deposition potential, (c) the role of operating frequency, and (d) the effect of pulse amplitude on peak current using SWASV. The graphs obtained using SWASV (3.6 μM Pb^2+^) in acetate buffer pH 3.0. Figure S2: Analytical results of Pb^2+^ detection in tap water samples by the proposed method. Figure S3: Analytical results of Pb^2+^ detection in sea water samples by the proposed method.
