# Supplementary material for: Design of Electrochemical Sensor Based on Pumpkin Peel Biomass-Derived Carbon Black-Modified Electrode for the Detection of Lead Ions
Source: Sensors (Basel). 2026 Feb 28;26(5):1524. doi: 10.3390/s26051524 (PMC12986631; doi:10.3390/s26051524)
Supplement: Supplementary file 1 [file sensors-26-01524-s001.zip › sensors-4067989-supplementary.pdf]

Article

Design of Electrochemical Sensor Based on Pumpkin Peel Biomass Derived Carbon Black Modified Electrode for the Detection of Lead Ions

Amal M. Aladwani [a], Esraa M. Bakhsh [a]*, Ekram Y. Danish [a], Zainab M. Hritani [a], Kalsoom Akhtar [a] and Sher Bahadar Khan [a]

[a]Chemistry Department, Faculty of Science, King Abdulaziz University, P. O. Box 80203, Jeddah 21589, Saudi Arabia.

*Correspondence: ibakhsh@kau.edu.sa

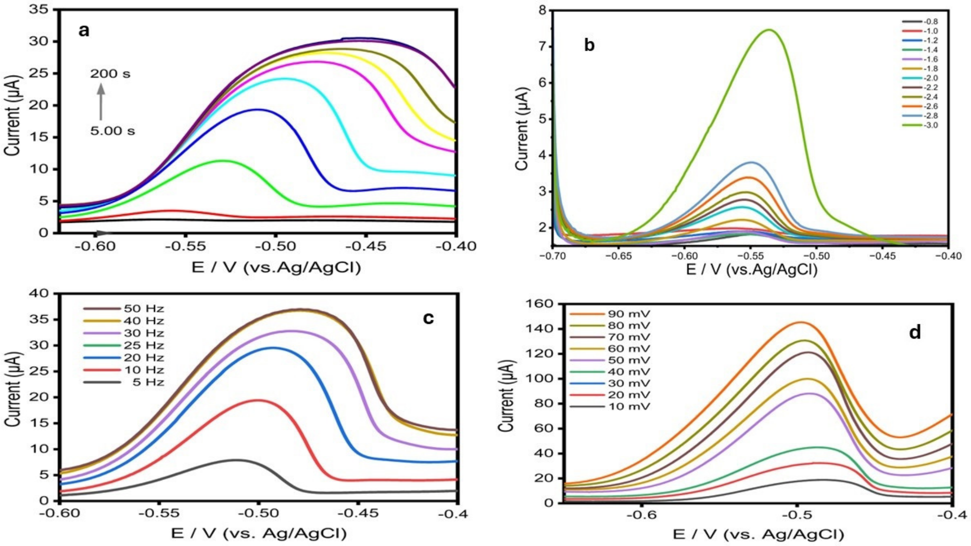

Figure S1. (a) The influence of deposition duration on peak current, (b) the impact of deposition potential, (c) the role of operating frequency, and (d) the effect of pulse amplitude on peak current using SWASV. The graphs obtained using SWASV (3.6 μM Pb^{2+}) in acetate buffer pH 3.0.

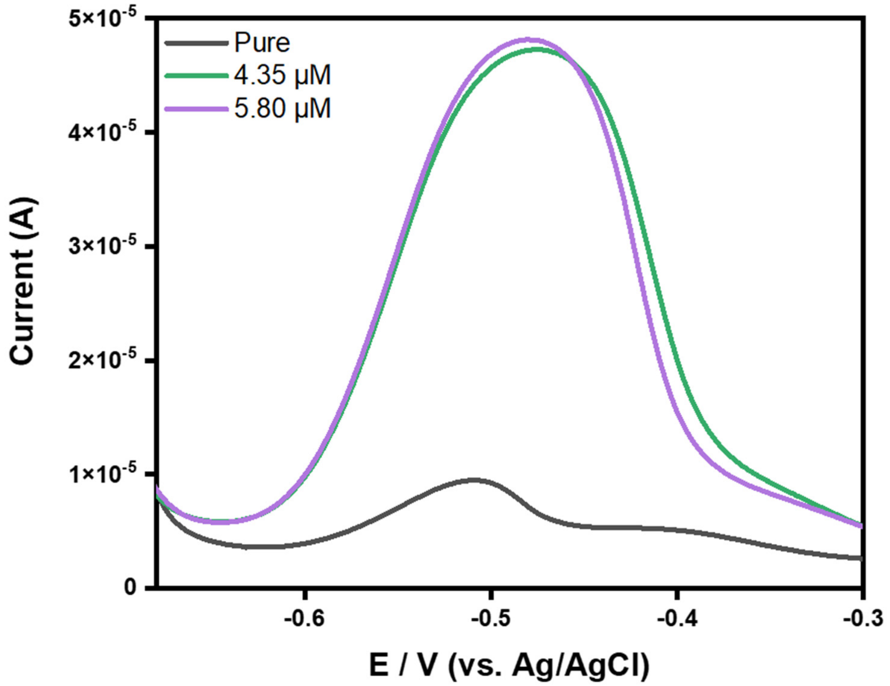

Figure S2. Analytical results of Pb^{2+} detection in tap water samples by the proposed method.

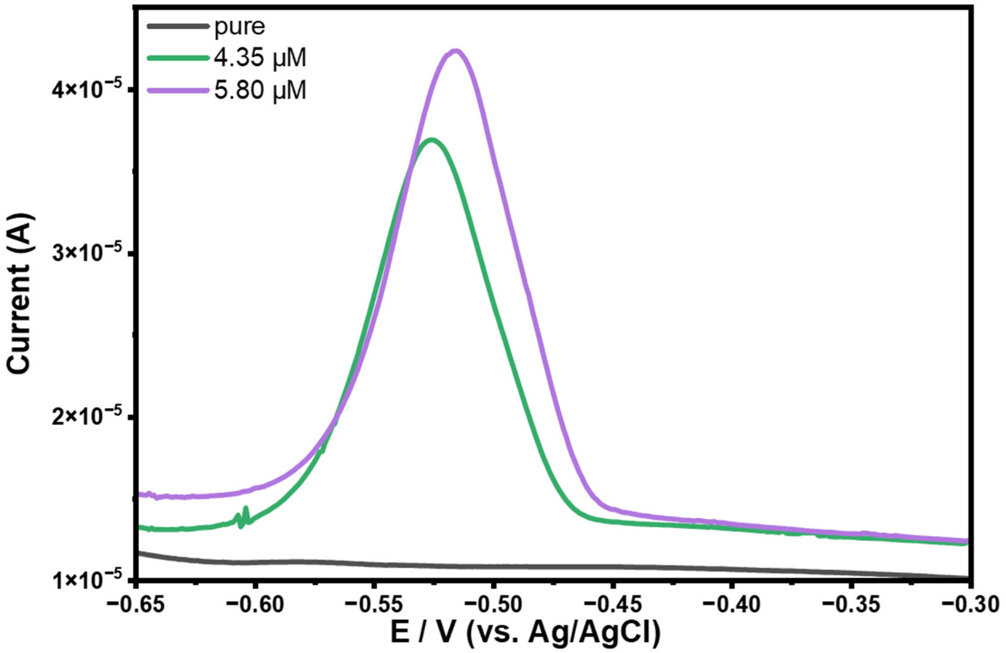

Figure S3. Analytical results of Pb^{2+} detection in sea water samples by the proposed method.
